# Ecologic Risk Factors for Infestation of *Rhipicephalus sanguineus* s.l. in a Rocky Mountain Spotted Fever-Endemic Area of Eastern Arizona

**DOI:** 10.4269/ajtmh.24-0485

**Published:** 2025-05-06

**Authors:** Maureen K. Brophy, Naomi A. Drexler, Nathan E. Stone, Joseph D. Busch, Rebecca Ballard, Reanna M. Bourgeois, Grant L. Pemberton, Christopher D. Paddock, Kalanthe Horiuchi, Brad J. Biggerstaff, Bessie H. Blocher, Gilbert J. Kersh, Harty Bendle, David M. Wagner, William L. Nicholson, Johanna S. Salzer

**Affiliations:** ^1^ Division of Vector-Borne Diseases, Centers for Disease Control and Prevention, Atlanta, Georgia;; ^2^ Division of Foodborne, Waterborne, and Environmental Diseases, Centers for Disease Control and Prevention, Atlanta, Georgia;; ^3^ Northern Arizona University Pathogen and Microbiome Institute, Flagstaff, Arizona;; ^4^ San Carlos Apache Tribe Health and Human Services, San Carlos, Arizona

## Abstract

Rocky Mountain spotted fever (RMSF) is a deadly tick-borne disease caused by the bacterium *Rickettsia rickettsii*. An ongoing epidemic of RMSF is affecting tribal communities in Arizona, with nearly 500 cases and 28 deaths since 2003. The San Carlos Apache Tribe has been consistently working to prevent RMSF using tick collars on dogs, pesticide treatments around homes, and increasing education for nearly a decade. Besides monitoring human disease levels and tick burden on dogs, we have little understanding of the long-term impact of prevention practices on tick abundance and infection rates in the peridomestic environment. We evaluated risk factors associated for tick infestation at home sites across the San Carlos Indian Reservation as well as *R. rickettsii* and *Rickettsia massiliae *prevalence in off-host ticks. Although the presence of fencing appears protective, the number of nearby structures is the most important risk factor associated with increased adult and nymphal tick abundance, highlighting the impact of a free-roaming dog population.

## INTRODUCTION

Rocky Mountain spotted fever (RMSF) is a potentially fatal tick-borne disease caused by the bacterium *Rickettsia rickettsii*. Throughout most of the United States, the bacterium is transmitted by *Dermacentor *species ticks, and historically, the disease has a case fatality rate as high as 20% in untreated patients.^1^ An ongoing epidemic of RMSF is affecting tribal communities in Arizona, with over 500 cases and 28 deaths since 2003.^2^ In Arizona, Mexico, and parts of South America, the bacterium is transmitted by *Rhipicephalus sanguineus *sensu lato, the brown dog tick. The San Carlos Apache Tribe has experienced ongoing RMSF since 2005, and the tribe’s public health response has been central to the development of evidence-based solutions for risk reduction and disease prevention methods used in other endemic areas.

*Rhipicephalus sanguineus *s.l. is a three-host metastriate tick found all over the world in peridomestic settings.^3^ Domesticated dogs are the preferred host in all life stages; however, the tick may parasitize other hosts, including humans. Dogs can experience high rates of tick infestation, with hundreds of *Rh. sanguineus* feeding on a single dog. Thus, areas with large populations of free-roaming dogs may experience large proportions of infestation in the dog population.^4^^–^^6^ Given the tick’s tendency for seeking refuge in a wide variety of materials in the peridomestic setting, *Rh. sanguineus* s.l. can survive harsh temperature extremes and may be active year-round in suitable climates.^3^

Determinants associated with increased risk of human RMSF cases have been studied across tribal communities in Arizona. Known risk factors for *Rh. sanguineus*-associated RMSF include large populations of free-roaming dogs (both owned and stray), high abundance of the tick vector, and positive *R. rickettsii* infection status in the canine or tick populations.^4^ RMSF is highly correlated with poverty,^7^^–^^10^ with associated risk factors being resource limitations, including low access to medical care, veterinary care, and solid waste disposal and limited community knowledge about tick-borne disease prevention.

In 2012, the San Carlos Apache Tribe and the CDC along with other state, federal, and nonprofit partners piloted an RMSF prevention project called the RMSF Rodeo. This prevention effort combined tick control on dogs, tick control around homes using environmental pesticides, and community education to reduce occurrence of RMSF. The project demonstrated reduction of ticks in the environment below detectable levels, reduction of ticks on community dogs, and a 43% reduction of human disease for 2 years after the end of the intervention,^4^ becoming the gold standard for *Rh. sanguineus-*associated RMSF prevention.^8^ The San Carlos Apache Tribe has continued to apply the integrated pest management practices for nearly a decade under the leadership of the San Carlos Animal Control Program. There have been no fatal RMSF cases reported on the San Carlos Apache Indian Reservation from 2018 through 2023, and total confirmed and probable cases on the reservation have been less than 10 cases per year in 2019–2023. Besides monitoring human disease levels and tick burden on dogs as part of treatment programs, there is little understanding of the long-term impact of prevention practices on RMSF risk, including factors associated with high tick burdens and prevalence of *R. rickettsii* infection in ticks.

## MATERIALS AND METHODS

The purpose of this assessment is to evaluate ecological factors associated with *Rh. sanguineus* abundance and the presence of *R. rickettsii* and *Rickettsia massiliae* in ticks within the San Carlos Apache Reservation. This assessment will help identify areas of risk and drivers for ongoing disease transmission in an endemic area with active integrated pest management programs.

### Site.

**Figure 1.**
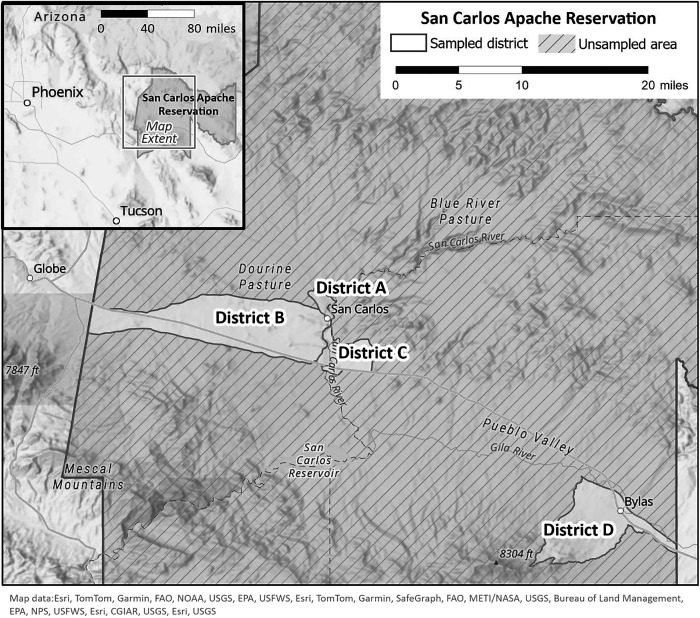
A population of just over 10,000 people is spread out across nearly 7,300 square kilometers that are divided into four housing districts. The inset shows the area surrounding the San Carlos Apache Reservation.

The San Carlos Apache Tribe is a sovereign indigenous nation in eastern Arizona, lying at the edge of the Sonoran Desert. A population of just over 10,000 people is spread out across nearly 7,300 square kilometers that are divided into four housing districts (Figure 1). The elevation ranges from 580 to 2,500 meters above sea level, with varied ecotypes.^11^ At the time of this study, the San Carlos Apache Tribe’s Animal Control Program used one animal control program manager, up to two full-time animal control officers, and one office assistant to provide animal wellness, animal bite response, and RMSF prevention services to the entire reservation.

### Tick collection.

A total of 100 traps were deployed every 2 months between March and September of 2022: 25 in each of the four districts. The twenty-five home sites per district were purposely chosen to represent areas of past human RMSF cases, high and low tick activity, and a broad geographic area based on geographic diversity (distance from a wash and high and low housing density) and recommendations from the San Carlos Apache Animal Control Program. Seven additional home sites were sampled in March based on tribal request. After the first trapping event, field staff attempted to return to home sites that were visited in the previous sampling effort; approximately 27% of home sites had to be replaced in subsequent collection events. To replace an enrolled home site, field staff used nearest neighbor replacement until a new home site was found.

Two dry ice-baited traps were set at each home site for approximately 2 hours in areas of each property appearing amenable to tick inhabitance, such as areas where dogs rest or shady, moist areas close to the home. Traps consisted of dry ice in lidded quart-sized plastic containers with two to three holes on each of the sides. Containers were then placed on an approximately 1-meter square white flannel cloth, with rocks or other heavy objects to weigh traps down from wind and to deter interference from dogs. During sampling events, data on temperature, humidity, wind speed, GPS coordinates, vegetation, and housing district and a description of the property (evidence of dogs on the property; the presence of a fence around property; the type of house structure building material; and tick harborage, including solid waste and woodpiles, observed) were also collected as well as the date and time of sample collections (temperature, wind speed, and vegetation data not presented). After 2 hours, traps were assessed for the presence of ticks, and temperature, humidity, and wind speed data collections were repeated. Ticks present on the traps were removed from the traps and stored in 70% ethanol. Ticks were later counted and morphologically identified to species, life stage, and sex (when applicable) in a laboratory setting. On traps with greater than 100 larval or nymphal ticks, counts were approximated.

Intervention efforts, including environmental pesticide application around homes and tick preventive application on dogs, led by the San Carlos Apache Animal Control continued to occur throughout the season as scheduled, with prevention campaigns scheduled directly after sampling events in March and September and a third campaign scheduled in June. Additionally, field teams in this project applied pesticide to homes identified during trapping that were infested with greater than 11 ticks to help protect residents against exposure to RMSF. Despite these public health control efforts, ticks continued to be collected in the four districts at each time point.

### *Rickettsia* detection.

Roughly 1,000 ticks per sampling event, primarily adults and nymphs, were selected for *R. rickettsii* and *R. massiliae *testing. *R. massiliae *is a spotted fever group *Rickettsia *species that has been detected in *Rh. sanguineus *populations throughout the world, including Arizona,^12^^,^^13^ and has been identified as the causative agent of mild flu-like illness in Europe, but to date, it has not been identified as pathogenic in the United States.^14^ Samples were tested from every home site where ticks were discovered, and adults and nymphs were prioritized; for most home sites, all ticks were processed. If adults or nymphs were not present at a home site, up to 100 larvae were pooled from that home site. Several home sites in July were so heavily infested that after ensuring representation from all home sites, we selected an even number of ticks for testing from these home sites.

Ticks collected during March were tested at CDC Rickettsial Zoonoses Branch in Atlanta, GA. Ticks were individually minced using sterile scalpels and then extracted according to the manufacturer’s instructions using a QIAamp DNA Mini Kit (Qiagen, Hilden, Germany). The samples were tested for *R. rickettsii* using a species-specific TaqMan polymerase chain reaction (PCR) assay that targets the 23S ribosomal RNA (rRNA) and a gene encoding hypothetical protein A1G_04230 as described by Kato et al.^15^

Ticks collected during the May through September sampling events were tested at Northern Arizona University’s Pathogen and Microbiome Institute. Ticks selected for testing from May through September were loaded into 96-well extractions plates for genomic DNA (gDNA) isolation. DNA extractions were processed in biosafety cabinets within a biosafety level 2 laboratory; a subset of DNA from each plate was tested for sterility. DNA quantity and quality were assessed for a subset of extractions by Nanodrop. All tick DNA extractions were also assessed for overall bacterial abundance using a modified version of the BactQuant 16S real-time PCR that targets a conserved region of the 16S rRNA gene^16^ and screened using a multiplexed real-time TaqMan PCR assay^17^ containing the RRi6^15^ and Rmass9666 targets.^18^ *Escherichia coli* was used as a positive template control. Sample cycle threshold (Ct) values less than reagent blank controls (the average Ct of four controls that were included on each extraction plate) were considered to contain adequate quantities of bacterial DNA to confidently conduct pathogen detection. A proficiency panel that included 10 blinded samples, some containing varying concentrations of *R. rickettsii* DNA, was used to confirm the equitability of the RRi6 assay’s performance across two institutions (CDC and Northern Arizona University). Genomic DNA from *R. rickettsii* was used as the positive template control for the RRi6/Rmass9666 TaqMan assay; Ct values <40 were considered positive. All samples were screened in triplicate.

## STATISTICAL ANALYSES

A multinomial regression model fitted with a bias-reducing estimation method was used to identify statistically significant factors associated with tick density by home site.^19^ The outcome measure, tick abundance, was a categorical index for adult and nymph abundance (0 ticks, 1–11 ticks, greater than 11 ticks) as quantifying the precise number of ticks was not worth the additional labor because it would not be an exact reflection on ticks present at the home site. Additionally, two logistic regression models were fit with a bias-reducing estimation method to identify predictors of 1) larval presence and 2) *R. rickettsii* infection in collected and tested ticks. For all models, backward variable selection was used, and odds ratios (ORs) and corresponding 95% CIs were calculated. Mean tick counts were compared across home sites using the Student’s *t*-test with the Welch approximation.

Variables commonly associated with tick infestations (either evidence based or anecdotally by tribal staff or community members), such as the presence of dogs, solid waste that can be used as tick harborage, woodpiles, proximity to a wash (waterways with intermittent flow in desert habitats), and presence of fences surrounding the property, were included in the models as predictors (Table 1). To assess housing density, ArcGIS was used to determine the number of structures greater than 450 square feet in size within a 100-foot radius of each home site using a Federal Emergency Management Agency dataset.^20^ Home sites that were within 250 feet of “large area” water bodies were identified using the U.S. Geological Survey National Hydrography Dataset^21^ to assess whether proximity to water sources, such as washes, impacted tick abundance. Data on pesticide application were retrospectively matched using SCAT Animal Control Program data. Data were analyzed using R Software (R Foundation for Statistical Computing, Vienna, Austria) with the “nnet” and “brglm2” packages.^22^^,^^23^ Statements of statistical significance are made at 5%.

**Table 1:** Variable list, type, and data source

Variable	Variable Type/Levels	Data Source
Adult/nymph abundance	Categorical (0, 1–11, >11)	Direct field observation
Larval presence	Dichotomous (yes, no)	Direct field observation
Month of collection	Nominal	Direct field observation
District	Nominal	Direct field observation
Dog presence	Dichotomous (yes, no)	Direct field observation
Harborage presence	Dichotomous (yes, no)	Direct field observation
Woodpile presence	Dichotomous (yes, no)	Direct field observation
Fencing presence	Dichotomous (yes, no)	Direct field observation
Proximity to wash	Dichotomous (<250, >250 ft)	U.S. Geological Survey
Nearby structures	Categorical (0, 1–3, 4+)	Federal Emergency Management Agency

## RESULTS

### Tick abundance.

**Figure 2.**
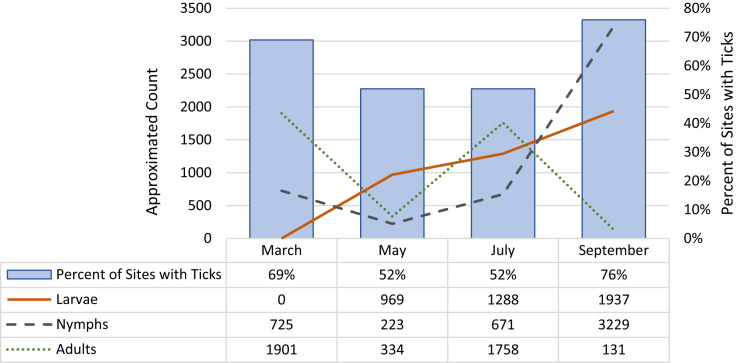
Percentage of sites with ticks and number of ticks by month.

Over 13,000 ticks were collected during the four sampling months combined. The average percentage of home sites that had one or more ticks over all four sampling events was 64%. District C had the highest percentage of home sites with one or more ticks over the entire sampling period; District D had the lowest percentage (Table 2). The highest percentage of home sites with ticks (76%) and the highest quantity of ticks (approximately 5,300) were collected in September (Figure 2; Table 2). There were two peaks in adult tick activity: in March and again, in July. Meanwhile, the number of nymphs dipped between March and May, and then, the number increased in both July and September. There were no larvae present during the March collection event, but estimated larval abundance increased in each subsequent collection event from May through September. During the remaining three collection events, all three life stages co-occurred.

**Table 2:** Number of home sites with one or more ticks (percentage of sampled home sites in the district)

Month	District				
	A	B	C	D	Average
March	16 (64%)	19 (76%)	19 (76%)	15 (60%)	17.5 (69%)
May	13 (52%)	18 (72%)	17 (68%)	7 (28%)	13 (52%)
July	16 (64%)	16 (64%)	14 (56%)	8 (32%)	13.5 (54%)
September	18 (72%)	19 (76%)	23 (92%)	16 (64%)	19 (76%)

### Risk factors for tick infestation.

A majority (89%) of sampled home sites had dogs present or an indication of dog presence, such as in-use food bowls or dog beds on the property. Fencing was present at 39% of home sites, although it was difficult to qualify how well the presence of a fence would exclude dogs from the property as fencing material and quality varied dramatically and were not recorded. Additionally, 62% and 43% of home sites were identified as having solid waste and woodpiles on the property, respectively.

Two separate multivariate regression models were fit to assess risk factors for tick abundance: one with adult and nymphal tick abundance as the outcome (Table 3) and the other with larval presence as the outcome (Table 4). Gravid *Rh. sanguineus *females can oviposit thousands of eggs in one location.^24^ Therefore, we did not explore larval abundance. Interpreting the results for both models in tandem is encouraged as larval presence indicates at least one gravid female tick present at the home site in recent weeks.^25^ For the first model, the number of nearby structures was a statistically significant predictor of higher tick abundance, where four or more nearby structures had an OR of 4.70 (95% CI: 2.21–10.00) for collecting 11+ adults and nymphs compared with zero ticks (Table 3). Higher tick abundance was less likely with the presence of a fence around the property (OR: 0.53, 95% CI: 0.29–0.96). Month was also a statistically significant predictor; as months went by, collecting higher numbers of adults and nymphs was less likely (Table 3). Larval presence was more likely in September compared with May (OR: 4.68, 95% CI: 2.53–8.89) and when dogs were present (OR: 3.47, 95% CI: 1.64–9.33). Having four or more structures nearby was also a statistically significant predictor of larval presence (OR: 3.52, 95% CI: 1.77–7.24) (Table 4).

**Table 3:** Final multinomial regression model for adult/nymphal tick abundance—odds ratio estimates and 95% CIs

Adult/Nymphal Tick Abundance	May vs. March	July vs. March	September vs. March	Fence Present vs. None	Nearby Structures 1–3 vs. 0	Nearby Structures 4+ vs. 0
1–10 vs. 0	0.55 (0.28–1.05)	**0.49 (0.24–0.98)**	0.92 (0.47–1.82)	0.63 (0.37–1.06)	1.45 (0.83–2.55)	**2.20 (1.20–4.01)**
11+ vs. 0	**0.24 (0.11–0.57)**	**0.38 (0.17–0.83)**	1.42 (0.70–2.90)	**0.53 (0.29–0.96)**	**2.88 (1.39–5.95)**	**4.70 (2.21–10.00)**

Statistically significant results are in bold.

**Table 4:** Final logistic regression model for larval presence—odds ratio estimates and 95% CIs

Larval Presence	July vs. May	September vs. May	Dog Present vs. None	Nearby Structures 1–3 vs. 0	Nearby Structures 4+ vs. 0
Yes vs. no	1.34 (0.72–2.53)	**4.68 (2.53–8.89)**	**3.47 (1.64–9.33)**	1.92 (0.98–3.85)	**3.52 (1.77–7.24)**

Statistically significant results are in bold.

### *Rickettsia* prevalence.

In total, 1.2% (*n* = 50) of the 4,218 ticks that were tested were positive for *R. rickettsii *(Table 5). Positive ticks were found at seven unique home sites (3% of home sites, including replacement home sites) across three districts over the four collection events, with one home site having positive ticks in two consecutive sampling collections. District B had positive home sites during all sample collections except for March. March had the lowest proportion of home sites and ticks testing positive, with only one tick from one home site in District A infected. Although July had the fewest number of home sites with tick infestations, it had the highest number of home sites with *R. rickettsii-*infected ticks; ultimately, 7.4% of home sites tested positive for the bacteria that month. The logistic regression model identified three statistically significant factors in infection rates: month, the presence of woodpiles, and life stage (larvae versus adults/nymphs). May, July, and September all had higher odds of infection than March (OR: 14.86, 95% CI: 2.81–78.75; OR: 7.99, 95% CI: 1.51–42.30; and OR: 14.60, 95% CI: 2.76–77.36, respectively). The presence of a woodpile on the site also had higher odds of infection (OR: 2.14, 95% CI: 1.20–3.84). Larvae had lower odds of infection than adults or nymphs (OR: 0.03, 95% CI: 0.002–0.44) (Table 6).

**Table 5:** *Rickettsia rickettsii* and *Rickettsia massiliae *testing results by month

Month	Home Sites Tested	Ticks Tested	*Rickettsia rickettsii*-Positive Home Sites,* n* (%)	*Rickettsia rickettsii*-Positive Ticks,* n* (%)	*Rickettsia massiliae*-Positive Home Sites,* n* (%)	*Rickettsia massiliae*-Positive Ticks,* n* (%)
March	69	1,000	1 (1.4)	1 (0.1)	N/A	N/A
May	55	740	1 (1.8)	16 (2.2)	0	0
July	54	1,352	4 (7.4)	16 (1.2)	0	0
September	76	1,036	2 (2.6)	17 (1.6)	1 (1.3)	64 (5.8)
Total	254	4,218	8 (3.1)*	50 (1.2)	1 (0.5)	64 (1.9)

N/A = not applicable.

* One home site was positive in both May and July. Seven unique home sites were positive over all months.

**Table 6:** Final logistic regression model for *Rickettsia rickettsii* infection—OR estimates and 95% CIs

Predictor	OR (95% CI)
May vs. March	14.86 (2.81–78.75)
July vs. March	7.99 (1.51–42.30)
September vs. March	14.60 (2.76–77.36)
Woodpile on site vs. none	2.14 (1.20–3.84)
Larvae vs. adults/nymphs	0.03 (0.002–0.44)

OR = odds ratio.

Sixty-four of 108 (59.2%) adult and nymphal ticks from a single home site in District D tested positive for *R. massiliae *in September; *R. massiliae *was not found at any other home sites or during any other sampling months.

## DISCUSSION

Although dogs are the primary host of *Rh. sanguineus*, dog presence was only a significant predictor of larval presence, not the abundance of adult and nymphal ticks. However, we encourage the interpretation of the two tick abundance models in tandem as larval presence indicates that at least one gravid female tick was present at the home site in the weeks leading up to trapping, likely having dropped off a dog. Additionally, although this study did not estimate dog density, a higher number of nearby structures was significantly associated with adult and nymphal tick abundance. This is likely an indication of larger populations of free-roaming dogs around areas with more densely concentrated dwellings, leading to higher tick abundance. The presence of free-roaming dogs is a known risk factor for RMSF transmission, and even homes with no evidence of “owned” dogs on the property are likely to be visited by roaming dogs. The relevance of free-roaming dogs with respect to tick abundance is also supported by the protective effect of fencing. Although the permeability of fencing varied across home sites and was not accounted for, the presence of a fence was protective against higher tick abundance. Therefore, the RMSF prevention strategy of targeted tick control on dogs remains critical in reducing human risk.^26^^–^^33^

Certain factors, such as housing material and proximity to washes, were included in this analysis because they were thought to play an important role in tick abundance but may not be as critical as previously thought. Although there was no significant difference in tick abundance by housing material, the mean count of ticks appeared higher for metal structures. It is not likely that the increased abundance was because of metal as a housing material, but rather, it was a result of the fact that metal homes in this area are typically trailers or other mobile homes on wheels or axles, with shaded space underneath where dogs are likely to rest and consequently, where ticks are likely to be present.

Local residents have suggested that there are more ticks at homes near washes, potentially because of the increased humidity in those areas and because free-roaming dogs use washes as corridors to travel. Our analysis found, however, that there was no significant difference in homes near water sources compared with those not near water sources. In fact, we found no difference in average humidity in home sites near water (27.8%) versus farther away (26.5%) either (difference = 1.3%, 95% CI: −4.4% to 7.1%). The caveat to a variable measuring linear distance between a trapping site and nearby water sources is that although a home site may be close to a water source “as the crow flies,” there may be significant elevation differences or other barriers between home sites and washes that were not evaluated or controlled for in this analysis.

This study tested a large number of off-host *Rh. sanguineus *ticks for *R. rickettsii *and *R. massiliae*. Ticks testing positive for *R. rickettsii* occurred sporadically with no clear pattern, except for the one repeated positive home site. The lack of a clear pattern may reflect the low prevalence of the bacteria and the fact that only subsets of ticks were tested from home sites with large numbers of ticks. Pathogen testing for ticks from the March sampling event occurred at a different facility with a different assay than the rest of the ticks, although given the sensitivity testing that was conducted, it is unlikely that this difference impacted results. In areas with endemic RMSF, *R. rickettsii *prevalence in the tick populations has been reported to range from 0.7% to 6.1%,^1^^,^^28^^,^^34^ suggesting that the 1.2% positivity rate in this study falls in the low to middle range of infected ticks. Cases of RMSF remain low on the San Carlos Apache Reservation, but it is clear that ongoing prevention efforts are still necessary.

We found an association between the presence of woodpiles at a household site and *R. rickettsii *infection in ticks. Although it may be possible that woodpiles act as protective harborage enough to protect infected ticks eclosing to the subsequent life stage, it is more likely spurious as we do not see the same association in woodpiles and tick abundance. Ticks spend most of their lives off the host in the environment, but we found no statistical difference in tick abundance owing to the presence of solid waste or woodpiles. However, data were not recorded on the volume, type, or proximity of solid waste to the home, so it is unclear whether these factors may have influenced the impact of these variables on the outcome. Considering the plethora of naturally occurring cracks, crevices, and other spaces for ticks to molt and oviposit, it is plausible that solid waste removal may not be as efficacious at reducing the risk of tick infestation as previously suggested.^1^ However, the presence of clutter near homes can impede proper pesticide application. Thus, reducing potential harborage sites remains an important part of integrated pest management against multiple types of arthropod pests and will continue to be recommended as part of tick management.

An integrated pest management approach to reducing tick abundance during times of high tick activity remains the most powerful approach to date for reducing risk of RMSF in endemic areas. However, additional novel approaches must be developed and considered. As the primary host for *Rh. sanguineus*, dogs are a critical intervention point to reducing community risk of RMSF in areas where it is circulating. Although applications of tick preventives, including collars, topicals, and oral medications, are effective, timely reapplication on free-roaming dogs can be a challenge. Hence, there is a critical need for funding and research into a canine vaccine against *Rh. sanguineus *ticks or *R. rickettsii*. Such a tool would be a monumental step toward breaking the zoonotic cycle of RMSF and limiting the impact of this devastating disease.^35^

## CONCLUSION

This evaluation of *Rh. sanguineus *ecology was the largest in recent history in Arizona and represents a long-forged collaboration by tribal, federal, and academic partners in the fight against RMSF. Results from this study may help inform evidence-based interventions not only in the San Carlos Apache Tribe but also, in other areas of the state and indeed, other countries, such as northern Mexico, where *Rh. sanguineus-*vectored RMSF is an ongoing challenge. Although there are many local hypotheses about factors related to tick infestations, these results demonstrate that housing density, month, and fencing are the most important predictors of tick abundance on the San Carlos Apache Reservation.
